# Nasal Polypi and Their Treatment

**Published:** 1898-02-12

**Authors:** 


### NASAL POLYPI AND THEIR TREATMENT.

Nasal polypi are met with so frequently in general practice and are in many cases so easily removed by very simple means that they are all too apt to be looked upon as trivial affairs. A post graduate lecture by Dr. Hunter Mackenzie[1] tends, however, to show on the one hand how often polypi exist without being discovered, and on the other how apt they are to recur unless they are much more effectually removed than is always possible by rough and ready methods.

In regard to diagnosis polypi in the posterior nares may not readily be seen, but as they hang down from the roof into the posterior and roomier part of the nose they are usually pedunculated and move freely on respiration, so that by making the patient close the opposite nostril and blow forcibly through the affected one the polypus will be distinctly observed to advance with expiration and not uncommonly to recede altogether out of sight during inspiration. The polypus, in fact, acts as a valve, usually permitting inspiration and impeding or entirely preventing expiration, and Dr. Mackenzie considers that a patient who presents this symptom is bound to have polypus in his posterior nares. Little polypi, or "buds," in the region of the middle turbinate body and the superior meatus of the nose are very apt to be overlooked, especially when situated behind cartilaginous or bony projections.

In the removal of the polypi this much may be affirmed, that while the forceps used in the ordinary way may suffice for the removal of large growths in roomy nostrils, they are quite ineffective where the nostrils are narrow and where the polypi are small, or in the not infrequent case of polypus buds. The cold snare, followed by the application of the electro-cautery to the point where the growth had been attached, is recommended for the larger and pendulous forms, but for those which are sessile, and for buds and the minute polypi which occur in the superior meatus, the curette is found the most effectual instrument. It must be remembered that in using the snare and the cautery, either general or local anæsthesia is required, and that the operation must be done with the aid of a speculum and strong reflected light, so that nothing shall be either snared or cauterised but what can be clearly seen.

Some points in diagnosis relating to the occurrence of pus and blood are worth remembering. It occasionally happens that polypi are found embedded in and surrounded by a copious purulent secretion which, after removal, rapidly re-forms and often has a putrefactive odour. This symptom points to implication of one or other of the accessory cavities of the nose.

Except when it arises from what is known as "bleeding polypus of the nose" hæmorrhage as a concomitant of mucus polypus of the nose is of bad significance and almost invariably indicates a high degree of malignancy. "Bleeding polypus of the nose," however, is a simple affair. It is of the nature of an angioma, and is attached to the anterior part of the septum. It is easily snipped off and its base touched with the electro-cautery, and as a rule it does not recur. This form usually affects women and, curiously enough, is almost invariably located in the left nostril.

[1] Lancet, Feb. 5, 1898.

